# Insights Into the Molecular Evolution of AT-Hook Motif Nuclear Localization Genes in *Brassica napus*

**DOI:** 10.3389/fpls.2021.714305

**Published:** 2021-09-09

**Authors:** Wei-Meng Zhang, Da Fang, Xiu-Zhu Cheng, Jun Cao, Xiao-Li Tan

**Affiliations:** School of Life Sciences, Jiangsu University, Zhenjiang, China

**Keywords:** *Brassica napus*, AT-hook motif, gene organization, gene duplication, *cis*-acting elements, selective pressure

## Abstract

AT-hook motif nuclear localization (AHL) proteins belong to a family of transcription factors, and play important roles in plant growth and development and response to various stresses through protein-DNA and protein-protein interactions. To better understand the *Brassica napus AHL* gene family, *AHL* genes in *B. napus* and related species were analyzed. Using *Arabidopsis* as a reference, 122 *AHL* gene family members were first identified in *B. napus*. According to the phylogenetic tree and gene organization, the BnaAHLs were classified into two clades (Clade-A and Clade-B) and three types (Type-I, Type-II, and Type-III). Gene organization and motif distribution analysis suggested that the *AHL* gene family is relatively conserved during evolution. These *BnaAHLs* are unevenly distributed on 38 chromosomes and expanded by whole-genome duplication (WGD) or segmental duplication. And large-scale loss events have also occurred in evolution. All types of *BnaAHLs* are subject to purification or neutral selection, while some positive selection sites are also identified in Type-II and Type-III groups. At the same time, the purification effect of Type-I members are stronger than that of the others. In addition, RNA-seq data and *cis*-acting element analysis also suggested that the BnaAHLs play important roles in *B. napus* growth and development, as well as in response to some abiotic and biotic stresses. Protein-protein interaction analysis identified some important BnaAHL-binding proteins, which also play key roles in plant growth and development. This study is helpful to fully understand the origin and evolution of the *AHL* gene in *B. napus*, and lays the foundation for their functional studies.

## Introduction

AT-hook motif nuclear localized (AHL) proteins contain two conserved structural units: the AT-hook motif and the plant and prokaryote conserved (PPC) domain. The latter is also annotated as the domain of un-known function #296 (DUF296) (Fujimoto et al., 2004). The AT-hook motif is a small DNA-binding motif that was first described as a high mobility group (HMG) non-histone chromosomal protein HMG-I/Y (Zhao et al., 1993). So far, it has been identified in various gene families of prokaryotes and eukaryotes, including the high mobility group A (HMGA) proteins of mammals (Aravind and Landsman, 1998). This AT-hook motif has an obvious feature: a conserved core sequence Arg-Gly-Arg amino acid (aa) with Arg-Lys or Pro residues on both sides. The AT-hook motif can use the conserved Arg-Gly-Arg residue to bind to the minor groove of stretches of AT-rich B-form DNA (Huth et al., 1997). Unlike most other transcription factors like leucine zipper and helix-loop-helix, AHLs mainly bind to the minor groove of duplex DNA (Aravind and Landsman, 1998; Reeves and Beckerbauer, 2001). When combined with DNA, the core sequence of Arg-Gly-Arg takes a concave conformation, and the side chains of two Arg residues should be firmly inserted into the minor groove, thereby modifying the chromatin structure (Huth et al., 1997). It is worth noting that the AT-hook motifs can recognize specific DNA structures rather than nucleotide sequences and activate or inhibit the transcription of many genes (Aravind and Landsman, 1998; Nagano et al., 2001). Proteins with PPC-like domains are also found in Bacteria and Archaea, but without AT-hook motifs (Fujimoto et al., 2004).

According to the conserved amino acid sequence, AT-hooks can be divided into two categories. AT-hooks containing Gly-Ser-Lys-Asn-Lys consensus sequences are Type-I motifs, and AT-hooks with Arg-Lys-Tyr consensus sequences at the end of the Arg-Gly-Arg core are Type-II motifs. The PPC/DUF296 conserved domain is located at the carboxy terminus of AHL proteins and contains about 120 aa (Fujimoto et al., 2004). According to sequence differences, PPC/DUF296 domain is divided into two types: PPC/DUF296 domain contains a core conserved sequence of Leu-Arg-Ser-His amino acids, which is defined as Type-A, while Type-B contains Phe-Thr-Pro-His in the upstream region (Zhao et al., 2014). All PPC/DUF296 domains also have a Gly-Arg-Phe-Glu-Ile-Leu conserved sequence, which is believed to mediate the interaction with transcription factors to promote the function of certain AHL proteins (Zhao et al., 2013).

Based on the evolution of *AHL* genes in land plants, they have been divided into two phylogenetic branches: Clades-A and Clades-B (Matsushita et al., 2007; Zhao et al., 2013, 2014). Clade-A has only one Type-I AT-hook motif and one intron-free Type-A PPC/DUF296 domain. Clade-B is further divided into two branches: one type of AHLs contains two AT-hooks (Type-I and Type-II) and a Type-B PPC/DUF296 domain; the other type of AHLs contain a Type-II AT-hook motif and a Type-B PPC/DUF296 domain (Zhao et al., 2014). In contrast to Clades-A, Clades-B contains introns (Bishop et al., 2020).

The *AHL* gene family is highly conserved in all land plants and play important roles in plant growth and development and stress response. Studies have shown that *AHL* gene family members can regulate a variety of developmental processes. *AHL18* promotes elongation and cell division in root apical meristem. Overexpression of *AHL18* makes the root system more developed, and promotes primary root growth (Sirl et al., 2020). An AT-hook protein, encoded by a palea defective mutant (*dp1*) of rice, can lead to a significant increase in the number of floral organs by affecting palea formation (Jin et al., 2011). Knockout of *AHL16* gene will activate various transposons and delay the flowering time in *Arabidopsis* (Xu et al., 2013a,b). Overexpression of the *AHL22* also delayed flowering by modifying *FLOWERING LOCUS T* (*FT*) chromatin in *Arabidopsis* (Xiao et al., 2009; Yun et al., 2012). *TRANSPOSABLE ELEMENT SILENCING VIA AT-HOOK (TEK*) gene can lead to male sterility by regulating the expression of *Arabinogalactan* (*AGP*) gene in anthers (Lou et al., 2014; Jia et al., 2015). *AHL21* is a direct target of AGAMOUS (AG). *AHL21* overexpression and loss of function can lead to reproductive defects (Ng et al., 2009). *Barren stalk fastigiate1* (*Baf1*) encodes an AT-hook DNA binding protein, which is a transcriptional regulator of maize panicle development. In *baf1* mutants, most plants failed to form panicles (Gallavotti et al., 2011). Overexpression of the *AHL22*, *AHL27*, and *AHL29* will suppress hypocotyl growth of *Arabidopsis* (Street et al., 2008; Xiao et al., 2009; Zhao et al., 2013). Two interacting AT-hook factors, AHL3 and AHL4, facilitate intercellular trafficking and regulate vascular tissue boundaries in *Arabidopsis* roots (Zhou et al., 2013). *ORE7/ESC* is the member of the *AHL* gene organization, which ectopic overexpression may extend leaf longevity by altering chromatin architecture in plants (Lim et al., 2007). *AHL15* gene is a suppressor of axillary meristems maturation in *Arabidopsis*, which can indirectly enhance the repressive effect on maturation of axillary meristems under short-day conditions, and prolong the lifespan of plant (Karami et al., 2020). In addition to regulating the growth and development of plants, some members of the *AHL* gene family also regulate the primary metabolism pathway including coordinating various responses to biotic and abiotic stresses in plants (Li and Kliebenstein, 2014). As an AT-hook protein, AGF1 can bind to the *cis*-elements of gibberellin and participate in its negative feedback regulation (Matsushita et al., 2007). Phosphorylation of *AHL10* coordinates drought stress and regulates jasmonic acid and auxin-related gene expression (Rashotte et al., 2003; Vom Endt et al., 2007; Wong et al., 2019). *AHL1* overexpression plants can better survive under drought, high salt and low temperature conditions (Zhou et al., 2016). The pathogen-associated molecular patterns (PAMPs)-triggered immunity (PTI) can resist the invasion of pathogenic microorganisms (Chisholm et al., 2006). This process will be inhibited by the over-expression of *AHL20*, thereby inhibiting the innate immune response (Lu et al., 2010). *AHL18* also plays important roles in disease resistance by forming homodimer or heterodimer in plant nucleus (Kumar et al., 2018).

Recently, *AHL* gene family has been identified and analyzed in several species. 29 *AHL* genes (*AtAHLs*) were identified from the *Arabidopsis* genome (Matsushita et al., 2007). The sorghum genome contains 22 *AHL* genes (Zhao et al., 2014). Twenty *AHL* genes have been discovered in the rice genome (Kim et al., 2011). Thirty-seven *AHL*s were identified from the maize genome (Bishop et al., 2020). Forty-eight, fifty-one, and ninety-nine *AHL*s were identified from three cottons (*Gossypium raimondii*, *G. arboretum*, and *G. hirsutum*), respectively (Zhao et al., 2020). The soybean genome contains 63 *AHL* genes (Wang et al., 2021b). About 7,500 years ago, a natural hybridization occurred between *Brassica rapa* (AA, 2*n* = 20) and *B. oleracea* (CC, 2*n* = 18), forming a natural polyploid *B. napus* (AACC, 2*n* = 38) (Chalhoub et al., 2014). *Brassica napus*, an important oil crop, has emerged as a model plant for studying genetics, evolution, and other biological processes. Rape yield is continually affected by multiple biotic and abiotic stresses. While *AHLs* are expected to play important and diverse roles in these stress responses, a detailed genome-wide analysis of *AHL* gene family has not been performed. In this study, we identified 122 *AHL* genes (*BnaAHLs*) from the *B. napus* genome and then analyzed their molecular evolution characteristics.

## Materials and Methods

### Genome-Wide Identification of AHL Proteins in the *B. napus* Genome

We obtain the *B. napus* genome data from the *B. napus* database.^1^ Twenty-nine AHLs were acquired from the *Arabidopsis* database.^2^ According to the protein sequence of AtAHLs, we searched homologous proteins in *B. napus* with homology comparison in blast+ (Camacho et al., 2009) with above 10^–5^ e-value. Based on the conserved domain of AHLs, the pfam (Finn et al., 2014) and CD-Search (Marchler-Bauer and Bryant, 2004) were also used to identify the DUF296 domain in the predicted BnaAHLs. Some candidate proteins that do not contain DUF296 domain will be discarded. Next, ExPASy (Gasteiger et al., 2003)^3^ was also used to investigate the physical and chemical properties of these AHL proteins. CELLO (Yu et al., 2006)^4^ was used to predict the location of these AHL proteins.

### Phylogenetic Analysis of AHL Proteins

The phylogenetic tree of the AHL proteins of *B. napus* and *Arabidopsis* was constructed using the neighbor-joining (NJ) method in MEGA 6.0 (Tamura et al., 2013)^5^ with the Poisson model and pairwise deletion. The reliability of phylogenetic tree was evaluated by 1,000 bootstrap duplications. At the same time, based on Jones Taylor Thornton (JTT) model and gamma distributed, we also constructed the phylogenetic tree using the maximum-likelihood (ML) method in MEGA 6.0. The phylogeny test is consistent with the NJ method, and other options are set to default values.

### Genes Structure Analysis

The AT-hook motifs of the BnaAHL proteins were predicted using the MEME v5.3.0^6^ (Bailey et al., 2015). The number of motifs should not exceed 7. The distribution of motifs occurs zero or one time in each sequence. In order to investigate the structural characteristics of the *BnaAHLs*, the gff3 file was downloaded from the *B. napus* database (see text footnote 1), which has the annotation information of the *B. napus* genome. The position information of introns and exons is obtained from the gff3 file. Meanwhile, the position information of the DUF296 conserved domain was also predicted with Batch CD-Search (Yang et al., 2020),^7^ and was submitted to TBtools^8^ (Chen et al., 2020) to graphically display gene structures and motif distributions.

### *BnaAHLs* Localization and Duplications

The annotation information data of *B. napus*, *Arabidopsis*, *B. rapa*, and *B. oleracea* was download from public databases (see text footnotes 1, 2),^9^ respectively. In order to study the duplication events and collinearity relationship of *B. napus*, MCScanX of the TBtools (Chen et al., 2020) was used to draw the collinearity map of *B. napus* and *B. napus*, *B. napus* and *Arabidopsis*, *B. napus* and *B. rapa*, and *B. napus* and *B. oleracea*, respectively.

Two genes on a branch at the end of the phylogenetic tree were regarded as a gene pair. Based on the coding DNA (CDS) sequence of these genes, the *Ks* value of the gene pairs can be calculated with TBtools (Chen et al., 2020). Based on the T = *Ks*/2λ, duplication time of these gene pairs was calculated by the commonly adopted clock-like rates (λ) of 1.4 × 10^–8^ synonymous substitutions per site per year (Wang et al., 2011).

### *Cis*-Acting Element Analysis

To identify the *cis*-acting element of *BnaAHLs*, TBtools (Chen et al., 2020) was used to obtain the 2,000 bp sequences in front of the genomic CDS. Then, the PlantCARE^10^ (Lescot et al., 2002) was used to predict the *cis*-acting elements on these promoters. Thus, the number and types of different *cis*-acting elements in *BnaAHLs* were classified and visualized with TBtools (Chen et al., 2020).

### Selective Pressure Analysis

Likelihood-ratio test was used to compare the implemented models. We used four evolution models [M8 (ωs ≥ 1), M8a (ωs = 1), M7 (beta), and M5 (gamma)] to evaluate the selective pressure (Yang et al., 2000). BnaAHL29e, BnaAHL13j, and BnaAHL13g proteins are used as query sequences to display the analysis results for Type-I, Type-II, and Type-III groups, respectively. All CDSs on each branch are used to calculate their selective pressure with the Selecton (Doron-Faigenboim et al., 2005).^11^ In order to determine the positions of these identified positive selection sites, I-TASSER (Yang et al., 2015)^12^ was used to predict the tertiary structures of these proteins.

### Gene Expression Analysis

The expression patterns of *BnaAHLs* in different tissues are obtained from the BnTIR (*Brassica napus* transcriptome information resource) database.^13^ RNA-seq data (accession number: GSE169299) of *B. napus* under the *Sclerotinia sclerotiorum* stress come from the GEO (Gene Expression Omnibus)^14^ (Clough and Barrett, 2016). Rape leaves were infected with *S. sclerotiorum*, and the epidermis (EPI), mesophyll (MES), and vascular (VAS) expression profiles were obtained. Next, RNA-seq data (accession number: GSE156029) of some abiotic stresses like drought and high temperature was also downloaded from the GEO (Clough and Barrett, 2016). All the RNA-seq data is standardized based on log2 scale, and clustered and visualized with TBtools (Chen et al., 2020).

### Protein-Protein Interaction Network Analysis

Interaction network of AHL proteins was predicted by the STRING^15^ database (Szklarczyk et al., 2019). Since there is no relevant data of *B. napus* in the STRING, we used the *B. rapa* data to reflect the interaction of AHL proteins in *B. napus*. All BnaAHLs proteins are used as the query to search for the interaction network of their homologous proteins in the cabbage. The network type is set to full network, that is, the edges indicate both functional and physical protein associations. The minimum required interaction score selects medium confidence (0.400). The number of interactors in the first shell cannot exceed 20, and that of the second shell cannot exceed 5.

## Results

### Identification and Classification of AHL Gene Family

122 AHL protein sequences were first identified in the *B. napus* genome, which contain conserved PPC/DUF296 domains and AT-hook motifs. Next, their physical and chemical properties were also analyzed (Table 1). These genes encode the amino acids ranging in length from 86 to 614 with the molecular weight from 9,580 to 66,979.04 Dalton. Isoelectric point (pI) of these amino acids ranged from 4.76 to 10.57. The hydrophilic coefficient of *BnaCnng02540D* is the largest (0.106), while that of *BnaAHL13c* is the smallest (−0.831). Approximately 98% of AHL transcripts were predicted to be located in the nucleus. This finding is consistent with one previous report that AHLs are nuclear localization proteins (Zhao et al., 2014).

**TABLE 1:** The information of *BnaAHL* gene family in *B. napus*.

Gene ID	Gene name	Genomic location	Protein length	Molecular weight	pI	GRAVY	Localization
BnaA01g02030D	BnaAHL25a	chrA01:1012289-1013197	302	30461.86	6.53	–0.328	Nuclear (3.539)
BnaA01g08690D	BnaAHL23a	chrA01:4167788-4168663	291	29312.56	5.8	–0.168	Nuclear (2.438)
BnaA01g08810D	BnaAHL13b	chrA01:4256372-4258249	407	43055.83	9.38	–0.724	Nuclear (4.312)
BnaA01g12480D	BnaAHL2c	chrA01:6242111-6243570	305	32152.98	9.54	–0.382	Nuclear (3.168)
BnaA01g12500D	BnaAHL24a	chrA01:6281168-6282145	315	33158.88	5.97	–0.655	Nuclear (4.201)
BnaA01g14710D	BnaAHL3a	chrA01:7405264-7407331	384	40761.97	4.96	–0.551	Nuclear (3.875)
BnaA01g33530D	BnaAHL14g	chrA01:22709076-22711461	386	40658.74	8.42	–0.815	Nuclear (4.346)
BnaA01g33550D	BnaAHL19a	chrA01:22718839-22719783	288	29600.83	6.05	–0.402	Nuclear (2.57)
BnaA02g18130D	BnaAHL29e	chrA02:10977792-10981133	288	29418.57	6.66	–0.453	Nuclear (3.387)
BnaA02g21540D	BnaAHL1b	chrA02:13882975-13884662	309	32596.18	9.71	–0.114	Nuclear (1.351), chloroplast (1.139)
BnaA02g33320D	BnaAHL6e	chrA02:23885893-23887885	401	42247.17	5.28	–0.511	Nuclear (3.122)
BnaA03g13050D	BnaAHL4a	chrA03:5941455-5943819	423	44542.24	5.54	–0.56	Nuclear (4.043)
BnaA03g15510D	BnaAHL10c	chrA03:7183138-7185596	262	27076.63	10.42	–0.323	Nuclear (3.16)
BnaA03g16760D	BnaAHL14h	chrA03:7833785-7835644	271	29011.34	6.83	–0.37	Nuclear (1.799)
BnaA03g19960D	BnaAHL16c	chrA03:9495040-9498578	510	56491.83	8.98	–0.493	Nuclear (3.352)
BnaA03g23240D	BnaAHL17b	chrA03:11090539-11091460	245	25914.31	9.1	–0.29	Nuclear (2.115)
BnaA03g43360D	BnaAHL23c	chrA03:21785206-21786081	274	27536.8	7.1	–0.136	Nuclear (2.178)
BnaA03g43460D	BnaAHL13e	chrA03:21841378-21844486	614	66979.04	5.59	–0.364	Nuclear (1.96), plasma membrane (1.771)
BnaA03g45520D	BnaAHL2b	chrA03:23168938-23170659	329	34243.19	9.14	–0.37	Nuclear (2.495)
BnaA03g45560D	BnaAHL24c	chrA03:23195615-23196598	317	33003.42	5.82	–0.672	Nuclear (4.071)
BnaA03g47290D	BnaAHL3d	chrA03:24231173-24234409	399	42364.63	5.34	–0.567	Nuclear (3.786)
BnaA04g00770D	BnaAHL11d	chrA04:545937-547458	335	35105.37	9.35	–0.421	Nuclear (4.14)
BnaA04g03490D	BnaAHL15f	chrA04:2372461-2374153	307	32180.7	5.77	–0.596	Nuclear (2.722)
BnaA04g05990D	BnaAHL20b	chrA04:4605495-4606334	279	28904.16	5.76	–0.334	Nuclear (2.612)
BnaA04g11020D	BnaAHL12a	chrA04:9545749-9548359	382	40595.36	9.17	–0.574	Nuclear (4.103)
BnaA04g19640D	BnaAHL10b	chrA04:15377291-15380137	375	39254.88	9.17	–0.484	Nuclear (3.805)
BnaA04g26180D	BnaAHL22a	chrA04:18688350-18689276	308	32386.02	6.67	–0.536	Nuclear (4.376)
BnaA04g26480D	BnaAHL9d	chrA04:18780555-18782668	341	35868.34	9.89	–0.432	Nuclear (4.332)
BnaA05g03180D	BnaAHL16b	chrA05:1722602-1723384	260	27207.98	9.4	–0.228	Nuclear (2.122)
BnaA05g04850D	BnaAHL18b	chrA05:2557538-2558767	310	32856.49	6.45	–0.558	Nuclear (4.139)
BnaA05g08700D	BnaAHL21d	chrA05:4817565-4818419	197	20268.38	5.67	–0.224	Nuclear (1.01), plasma membrane (1.109), extracellular (1.049)
BnaA05g32890D	BnaAHL19c	chrA05:22454433-22455377	314	32179.71	5.75	–0.412	Nuclear (3.404)
BnaA06g09360D	BnaAHL28a	chrA06:5024685-5025302	205	21241.16	8.49	–0.121	Nuclear (1.744), plasma membrane (1.102)
BnaA06g13280D	BnaAHL13f	chrA06:6958604-6959756	262	26655.67	8.76	–0.322	Nuclear (2.14)
BnaA06g13290D	BnaAHL13g	chrA06:6962286-6963512	270	27271.34	9.3	–0.344	Nuclear (2.586)
BnaA06g13300D	BnaAHL13h	chrA06:6964906-6966148	303	31188.96	10.05	–0.388	Nuclear (3.973)
BnaA06g13310D	BnaAHL13i	chrA06:6968272-6969569	311	32331.13	7.01	–0.448	Nuclear (3.185)
BnaA06g14760D	BnaAHL27c	chrA06:7997603-7998538	274	28874.79	6.49	–0.739	Nuclear (3.901)
BnaA06g21720D	BnaAHL6a	chrA06:15160112-15162755	403	42146.13	8.64	–0.508	Nuclear (3.083)
BnaA06g39570D	BnaAHL14a	chrA06_random:1621067-1623681	372	38837.75	8.72	–0.667	Nuclear (4.715)
BnaA07g11200D	BnaAHL27a	chrA07:10515217-10516149	272	28475.11	6.3	–0.783	Nuclear (4.098)
BnaA07g21350D	BnaAHL29c	chrA07:16568777-16569640	270	28042.93	6.22	–0.621	Nuclear (3.304)
BnaA07g32820D	BnaAHL29a	chrA07:22634449-22635315	280	28801.81	6.54	–0.497	Nuclear (3.549)
BnaA07g37810D	BnaAHL15b	chrA07_random:1304362-1305955	298	31257.55	5.76	–0.52	Nuclear (2.374)
BnaA07g38060D	BnaAHL9a	chrA07_random:1451125-1452616	328	34916.25	9.15	–0.37	Nuclear (4.189)
BnaA08g08510D	BnaAHL13c	chrA08:8337686-8339718	440	46074.73	9.46	–0.831	Nuclear (3.551)
BnaA08g21560D	BnaAHL27e	chrA08:15925517-15926452	301	31336.54	6.56	–0.639	Nuclear (3.761)
BnaA09g05970D	BnaAHL6d	chrA09:2903525-2905581	392	41453.46	8.76	–0.558	Nuclear (3.531)
BnaA09g10970D	BnaAHL5b	chrA09:5647428-5649660	378	39570.29	9.11	–0.519	Nuclear (4.355)
BnaA09g12490D	BnaAHL5c	chrA09:6620892-6623182	367	38473.28	8.51	–0.477	Nuclear (3.874)
BnaA09g18090D	BnaAHL8b	chrA09:11182638-11184564	353	37749.9	7.94	–0.577	Nuclear (4.156)
BnaA09g39090D	BnaAHL11b	chrA09:27757324-27759265	344	35868.88	9.18	–0.58	Nuclear (4.519)
BnaA09g55070D	BnaAHL15c	chrA09_random:3033021-3034593	297	31097.24	5.28	–0.569	Nuclear (2.941)
BnaA10g07850D	BnaAHL4c	chrA10:6372527-6375412	429	45488.21	5.34	–0.598	Nuclear (4.04)
BnaAnng11530D	BnaAHL14d	chrAnn_random:12510503-12513033	349	36573.22	7.89	–0.722	Nuclear (4.644)
BnaAnng13510D	BnaAHL23b	chrAnn_random:14506055-14507405	292	29307.45	5.8	–0.17	Nuclear (2.592)
BnaAnng15660D	BnaAHL21a	chrAnn_random:16675647-16676507	286	29304.55	6.21	–0.267	Nuclear (2.772)
BnaAnng20150D	BnaAHL4f	chrAnn_random:21918514-21920931	419	43957.41	5.04	–0.515	Nuclear (3.562)
BnaAnng26620D	BnaAHL13j	chrAnn_random:30539232-30540459	297	30970.61	6.08	–0.394	Nuclear (3.569)
BnaAnng35660D	BnaAHL16f	chrAnn_random:40391305-40392090	261	27674.4	9.6	–0.408	Nuclear (3.586)
BnaC01g03170D	BnaAHL25b	chrC01:1628472-1629380	302	30477.88	6.6	–0.333	Nuclear (3.678)
BnaC01g10490D	BnaAHL13a	chrC01:6233583-6235488	420	44050.75	9.37	–0.749	Nuclear (4.29)
BnaC01g14210D	BnaAHL2d	chrC01:9452185-9453468	215	22763.48	8.97	–0.424	Nuclear (3.388)
BnaC01g14280D	BnaAHL24b	chrC01:9545116-9546093	315	33162.87	5.97	–0.652	Nuclear (4.232)
BnaC01g17270D	BnaAHL3b	chrC01:11819819-11821850	378	40255.58	5.52	–0.563	Nuclear (3.711)
BnaC01g40030D	BnaAHL14f	chrC01:38410679-38412955	381	39914.95	8.82	–0.769	Nuclear (4.47)
BnaC01g40050D	BnaAHL19b	chrC01:38425385-38426332	289	29711.03	6.44	–0.376	Nuclear (2.431)
BnaC02g15620D	BnaAHL4e	chrC02:11228173-11230283	419	44292.88	5.3	–0.581	Nuclear (3.867)
BnaC02g24120D	BnaAHL29f	chrC02:21369707-21370570	271	27871.87	6.59	–0.49	Nuclear (3.424)
BnaC02g28560D	BnaAHL1a	chrC02:27253896-27257645	268	28251.97	9.59	–0.283	Nuclear (2.415)
BnaC02g28570D	BnaAHL26	chrC02:27360192-27361175	327	34541.56	7.23	–0.624	Nuclear (4.423)
BnaC02g39430D	BnaAHL14c	chrC02:42337986-42340353	356	37397.19	8.65	–0.701	Nuclear (4.658)
BnaC03g15920D	BnaAHL4b	chrC03:8043866-8046192	424	44442.23	5.57	–0.506	Nuclear (3.967)
BnaC03g18730D	BnaAHL10d	chrC03:9602635-9605143	262	26997.57	10.57	–0.29	Nuclear (2.934)
BnaC03g19350D	BnaAHL21b	chrC03:10055072-10055893	273	27945.32	6.5	–0.166	Nuclear (2.03)
BnaC03g20230D	BnaAHL14i	chrC03:10593676-10595194	263	28441.7	8.61	–0.393	Nuclear (1.434), extracellular (1.238)
BnaC03g23960D	BnaAHL16d	chrC03:13408102-13411567	510	56551.07	8.98	–0.484	Nuclear (3.605)
BnaC03g27460D	BnaAHL17a	chrC03:15943017-15943947	245	25806.17	8.84	–0.273	Nuclear (2.049)
BnaC03g51800D	BnaAHL6b	chrC03:36477962-36480539	399	41890.72	8.64	–0.543	Nuclear (3.297)
BnaC04g04190D	BnaAHL18a	chrC04:3183022-3184039	309	32693.36	6.36	–0.546	Nuclear (4.233)
BnaC04g09840D	BnaAHL21e	chrC04:7432188-7433030	192	19773.91	5.52	–0.151	Plasma membrane (1.199), extracellular (1.316)
BnaC04g21860D	BnaAHL11c	chrC04:22939026-22940661	338	35600.99	9.35	–0.406	Nuclear (3.915)
BnaC04g25370D	BnaAHL15e	chrC04:26456222-26457145	307	32259.85	5.72	–0.559	Nuclear (2.56)
BnaC04g26040D	BnaAHL14e	chrC04:27334997-27337369	410	44185.73	8.96	–0.512	Nuclear (4.081)
BnaC04g28620D	BnaAHL20a	chrC04:30013644-30014492	282	29171.41	5.84	–0.35	Nuclear (2.58)
BnaC04g33040D	BnaAHL12b	chrC04:34729503-34731881	382	40645.43	9.17	–0.583	Nuclear (4.135)
BnaC04g44020D	BnaAHL10a	chrC04:44217405-44221510	398	41427.52	9.82	–0.375	Nuclear (3.34)
BnaC04g48590D	BnaAHL16e	chrC04:47129064-47129849	261	27607.33	9.35	–0.338	Nuclear (3.379)
BnaC04g50180D	BnaAHL22b	chrC04:47990679-47992087	312	32649.24	6.49	–0.546	Nuclear (4.374)
BnaC04g50510D	BnaAHL9c	chrC04:48147182-48149642	349	36623.08	9.89	–0.455	Nuclear (4.233)
BnaC04g52840D	BnaAHL16a	chrC04_random:567578-568660	260	27238	9.4	–0.238	Nuclear (2.189)
BnaC05g14850D	BnaAHL13k	chrC05:8762705-8763880	266	27126.97	4.76	–0.375	Nuclear (2.932)
BnaC05g14860D	BnaAHL13l	chrC05:8763947-8766450	248	25562.89	9.59	–0.152	Nuclear (2.059)
BnaC05g16200D	BnaAHL27d	chrC05:9956800-9957726	269	28416.23	6.45	–0.769	Nuclear (3.921)
BnaC05g49680D	BnaAHL28b	chrC05_random:443514-445290	275	29242.98	9.59	–0.464	Nuclear (3.441)
BnaC06g21820D	BnaAHL29d	chrC06:23995353-23996222	272	28478.47	5.97	–0.613	Nuclear (3.405)
BnaC06g37300D	BnaAHL29b	chrC06:35395936-35396808	276	28089.08	6.49	–0.461	Nuclear (3.183)
BnaC07g15080D	BnaAHL27b	chrC07:21040522-21041457	273	28788.47	6.56	–0.824	Nuclear (4.121)
BnaC07g16150D	BnaAHL28c	chrC07:22130806-22132893	305	34128.18	5.75	–0.09	Cytoplasmic (1.017), extracellular (1.092)
BnaC07g34930D	BnaAHL13m	chrC07:37455368-37456678	259	26705.88	5.8	–0.285	Nuclear (3.467)
BnaC07g34960D	BnaAHL13n	chrC07:37469199-37470502	301	31276.58	9.01	–0.527	Nuclear (3.97)
BnaC07g37560D	BnaAHL2a	chrC07:39243169-39244832	329	34287.24	9.14	–0.389	Nuclear (2.685)
BnaC07g37620D	BnaAHL24d	chrC07:39305131-39306114	327	34431.98	6.3	–0.721	Nuclear (4.202)
BnaC07g39510D	BnaAHL3c	chrC07:40320219-40322011	396	42160.44	5.33	–0.576	Nuclear (3.765)
BnaC07g49380D	BnaAHL14b	chrC07_random:1919401-1922012	368	38394.32	9.07	–0.645	Nuclear (4.661)
BnaC08g11120D	BnaAHL13d	chrC08:16541191-16543280	435	45745.41	9.46	–0.829	Nuclear (3.575)
BnaC08g19580D	BnaAHL27f	chrC08:22435952-22436902	292	30683.74	6.7	–0.732	Nuclear (3.985)
BnaC08g26470D	BnaAHL15d	chrC08:27867014-27868576	295	30802.96	5.39	–0.526	Nuclear (2.966)
BnaC08g31280D	BnaAHL11a	chrC08:30721169-30723200	344	36021.19	9.32	–0.545	Nuclear (4.152)
BnaC09g05560D	BnaAHL6c	chrC09:3260077-3262538	391	41063.89	8.4	–0.54	Nuclear (3.498)
BnaC09g11250D	BnaAHL5a	chrC09:7871395-7873527	370	38724.43	9.2	–0.538	Nuclear (3.943)
BnaC09g19530D	BnaAHL8a	chrC09:16356122-16357717	358	38279.48	9.39	–0.641	Nuclear (4.353)
BnaC09g28030D	BnaAHL4d	chrC09:30082464-30085481	439	46800.57	9.49	–0.453	Nuclear (3.568)
BnaCnng02530D	BnaAHL7a	chrCnn_random:2197797-2199572	316	33474.95	9.17	–0.322	Nuclear (2.781)
BnaCnng02540D	BnaAHL7b	chrCnn_random:2199636-2202285	231	24531.97	5.69	0.106	Plasma membrane (3.231)
BnaCnng14340D	BnaAHL16g	chrCnn_random:13175770-13176327	166	18351.95	9.46	–0.302	Nuclear (2.1)
BnaCnng14360D	BnaAHL16h	chrCnn_random:13271386-13271794	86	9580	10.39	–0.513	Nuclear (1.414), mitochondrial (1.705), extracellular (1.035)
BnaCnng20900D	BnaAHL9b	chrCnn_random:19622175-19623690	340	35730.21	9.51	–0.32	Nuclear (4.184)
BnaCnng36000D	BnaAHL15a	chrCnn_random:34553327-34554925	296	31129.46	5.9	–0.51	Nuclear (2.219)
BnaCnng43040D	BnaAHL5d	chrCnn_random:42167424-42169399	362	38011.68	9.05	–0.479	Nuclear (3.912)
BnaCnng44570D	BnaAHL19d	chrCnn_random:43703451-43704386	311	31922.5	5.75	–0.392	Nuclear (3.397)
BnaCnng77890D	BnaAHL21c	chrCnn_random:79427531-79428352	273	27923.27	6.35	–0.158	Nuclear (1.896)

**FIGURE 1:**
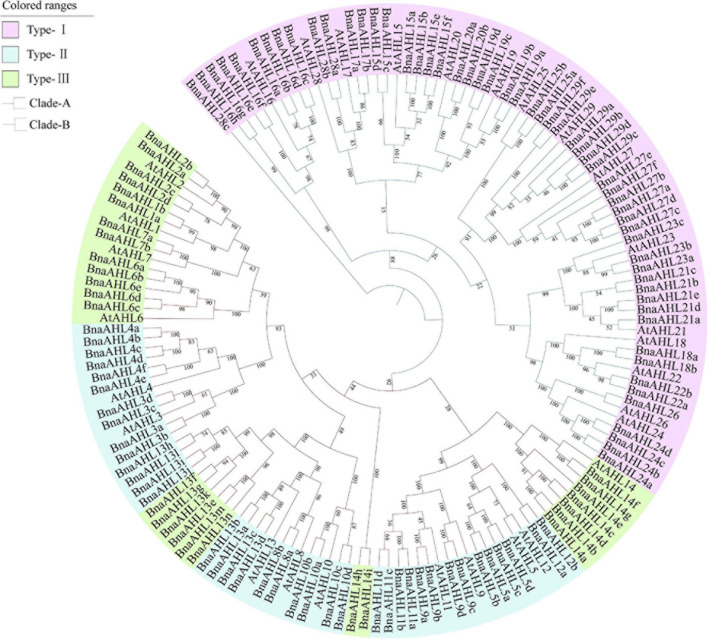
Phylogenetic relationship of *AHL* genes in *B. napus* and *Arabidopsis*. Branches of different colors indicate different evolutionary clades. Each branch has a number indicating the percentage of reliability of the evolutionary branch.

In order to understand the evolutionary relationship of AHLs in *B. napus* and *Arabidopsis*, their protein sequences were alignmented with Clustal X (Jeanmougin et al., 1998) and phylogenetic trees were constructed by ML and NJ methods. The evolutionary relationship between the ML tree and the NJ tree is very similar (Figure 1 and Supplementary Figure 1). It showed that *AHLs* can be clearly divided into two clades: Clade-A and Clade-B. Clade-A contains 56 *AHL* genes of *B. napus* and 15 *AHL* genes of *Arabidopsis*, and it is also classified into Type-I. There are 80 *AHL* genes in Clade-B, 66 of which are from the *B. napus* and other 14 are from the *Arabidopsis*. Clade-B is further divided into two types: Type-II and Type-III, of which Type-II contains 47 *AHL* genes, while Type-III includes 33 *AHL* genes (Figure 1).

### Gene Structures and Motif Analysis of *AHLs* in *B. napus*

**FIGURE 2:**
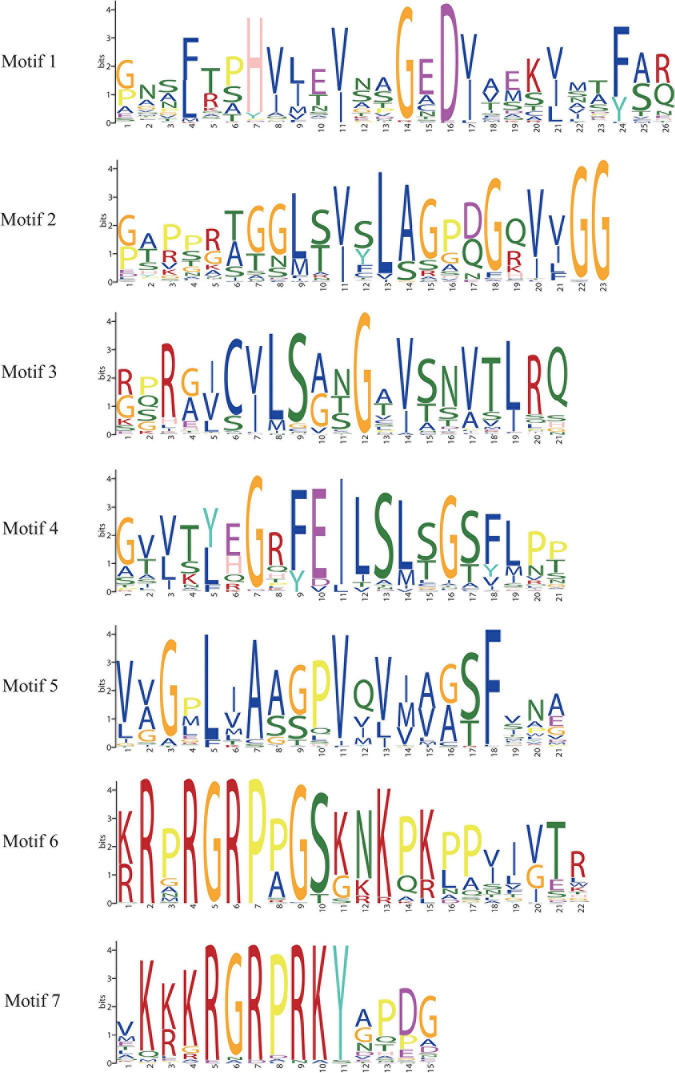
Sequence logos of conserved motifs in *BnaAHLs*. The logo indicates the sequence of seven motifs. A letter represents an amino acid, and the size represents its conservation at this position.

In order to study the homology domain and conservation degree of the *BnaAHLs*, MEME (Bailey et al., 2015) and TBtools (Chen et al., 2020) were used to predict and visualize their conserved domain and gene organization, respectively. As a result, 7 conserved motifs were found in the 122 AHLs of *B. napus* (Figure 2). Motif 6 and motif 7 have highly conserved R-G-R-P and R-G-R-P-R-K-Y amino acid sequences, respectively. Among them, R-G-R-P is the core sequence shared by the two motifs (Figure 2). This finding is consistent with previous reports of the AHL structure (Zhao et al., 2014, 2020; Bishop et al., 2020; Wang et al., 2021a).

**FIGURE 3:**
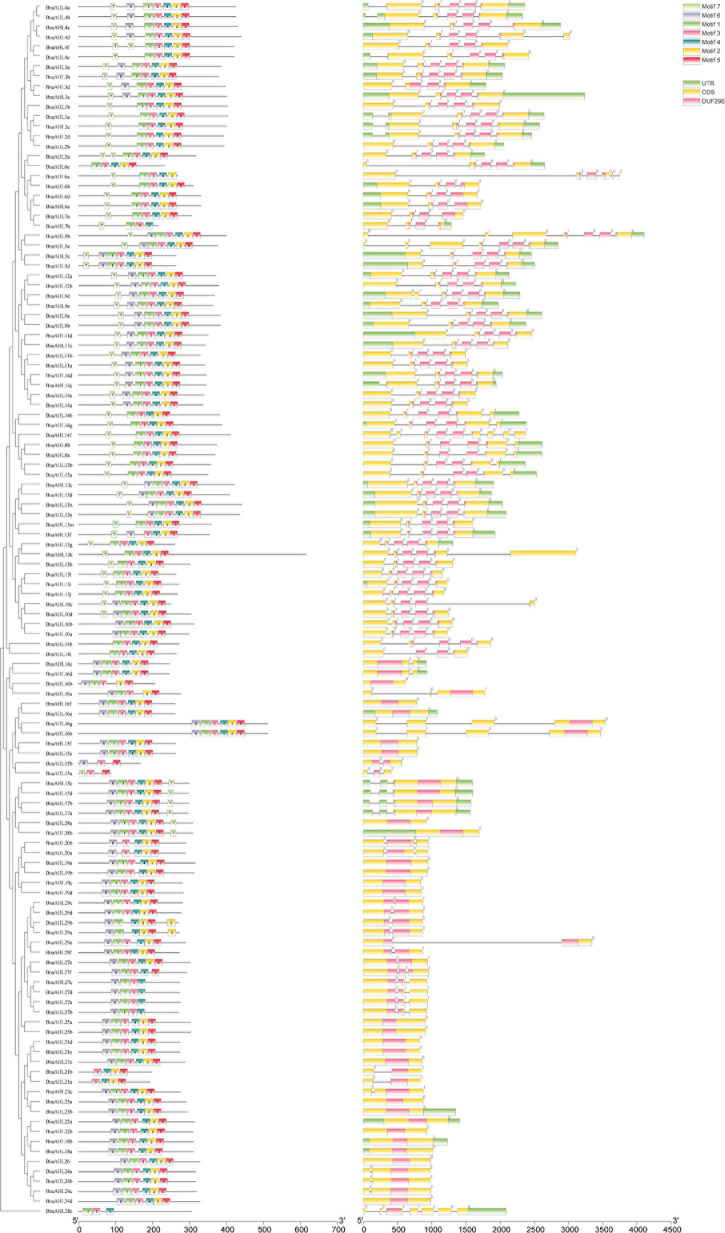
Motif distribution and exon-intron structure of *AHLs*. The neighbor-joining phylogenetic tree was constructed with MEGA 6.0 software, and the conserved motifs and gene structure were predicted with MEME and Tbtools, respectively. The scale at the bottom indicates the length of protein and DNA sequences, respectively. In the left picture, different colored boxes indicate different conserved motifs, and the black line indicates non-conserved amino acids. The black line on the right represents introns, the green box represents untranslated 5′ and 3′ regions; the yellow box represents exons, and the purple box represents the conserved domain of the *AHLs*.

The appearance of introns can enhance the transcriptional activation of *AHLs* (Zhao et al., 2014). We also investigated the distribution of introns and exons to study the diversity of gene structure. The structure of *AHLs* can be clearly divided into two types: one contains multiple introns, and the other contains no or only one intron (Figure 3). The 66 genes in Clade-B have 3 introns, except for the 14 genes with 1 intron in Clade-A, the other 42 *BnaAHLs* without intron. While in other species, *AHLs* in Clade-A do not contain any intron (Zhao et al., 2014, 2020; Bishop et al., 2020).

In addition, we also found that the intron phases of *BnaAHLs* were highly conserved in the same clade, implicating the evolutionary similarity between these members. There are phase 0, 1, and 2 introns in all eukaryotic genomes, and the 0 phase intron accounts for the highest proportion and has the highest level of conservation (Long et al., 1995). The PPC/DUF296 domains of most *AHL* genes in Clade-A doesn’t have any intron, while 14 *AHL* genes have phase 0 introns. However, the PPC/DUF296 conserved domain of most *AHL* genes in Clade-B has the same intron phase distribution except *BnaAHL1a*, *BnaAHL1b*, *BnaAHL2a*, *BnaAHL2b*, *BnaAHL2c*, and *BnaAHL2d*. This result implies that the AHL gene family has a relatively conservative evolutionary relationship in each clade (Figure 3).

### Chromosomal Distribution and Duplication of *BnaAHLs*

**FIGURE 4:**
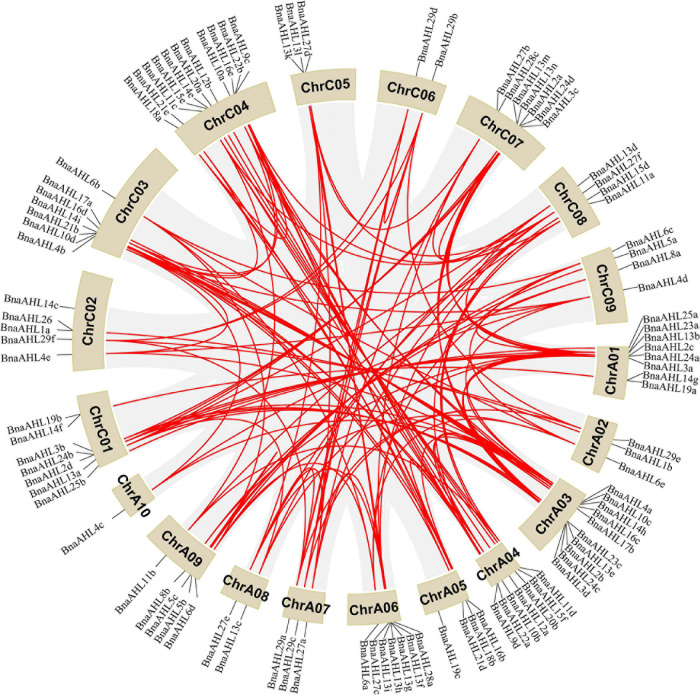
Distribution of *BnaAHLs* on chromosome. One hundred *BnaAHLs* are distributed on nineteen different chromosomes of *B. napus*. In addition, 22 *BnaAHLs* are not mapped to any chromosomes. The gray lines in the background indicate the collinear blocks in the genomes of *B. napus*, while the red lines emphasize the segmental duplication of the *AHLs*. The brown block indicates the chromosome and its length represents the size of the chromosome.

The A and C genomes of Brassica are not only highly conserved, but also characterized by a large number of gene loss, tandem duplication, polyploidy, and chromosome rearrangement (Struss et al., 1996; Howell et al., 2008; Mun et al., 2009; Liu et al., 2014). Genomic mapping of the *BnaAHLs* showed that 100 *BnaAHLs* were unevenly distributed on 38 chromosomes (Figure 4), while the other 22 genes were not accurately located. Among them, 50 *BnaAHLs* are distributed on the A and C subgenome, respectively. A large number of *BnaAHL* genes were distributed in chromosome ChrC04 and ChrA03, which contained 11 and 10 members, respectively. However, only one member was found in ChrA10.

About 20 million years ago, *Arabidopsis* diverged from Brassica, a member of the cruciferous family (Yang et al., 1999). The whole Brassica genome triploidized about 13 million years ago (Yang et al., 1999; Lysak et al., 2005). The divergence time of A and C genome fragments in the diploid ancestors of *B*. *napus* spanned about 0.12–1.37 million years (Cheung et al., 2009). The common mutation rate of 1.4 × 10^–8^ synonym substitutions per site per year was used to estimate the time of BnaAHLs divergence (Wang et al., 2011). We found that these *BnaAHLs* had been duplicated about 0.63–6.55 million years ago (Supplementary Table 1). Among them, seven gene pairs (*BnaAHL8b/BnaAHL8a*, *BnaAHL13i/BnaAHL13j*, *BnaAHL23a/BnaAHL23b*, *BnaAHL15b/BnaAHL15a*, *BnaAHL19a/BnaAHL 19b*, *BnaAHL16f/BnaAHL16e*, *BnaAHL29e/BnaAHL29f*) are duplicated during the divergence of A and C genomes, while the other gene paires duplicated after triploidy of the whole genome, but before the divergence of A and C genomes. Therefore, whole genome duplication is the main reason of the *BnaAHLs* amplification. It can be seen from Supplementary Table 1 that the two *BnaAHLs* in a gene pair are from the A and C genomes, respectively. So it was further illustrated that the amplification of *BnaAHLs* is also caused by polyploidization. Among them, *BnaAHL27e* and *BnaAHL27f* were duplicated about 6.12 million years ago. The type of replication of some *BnaAHLs* is tandem duplication (Supplementary Table 2). In additional, *BnaAHL13f*, *BnaAHL13g*, *BnaAHL13h*, and *BnaAHL13i* are distributed in tandem on chromosomes (Figure 4 and Table 1), which is further confirmed that the *BnaAHL* genes have duplicated in tandem during the evolution.

**FIGURE 5:**
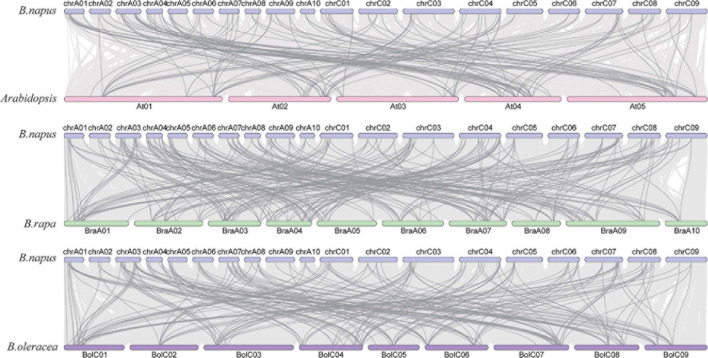
The collinear relationship of *AHLs* on chromosome. The blue, pink, green, and purple bars represent the chromosomes of *B. napus*, *Arabidopsis*, *B. rapa*, and *B. oleracea*, respectively. The gray lines in the background indicate the collinear blocks in the genomes of *B. napus* and *Arabidopsis*, *B. rapa*, and *B. oleracea*, while the darker colored lines emphasize the segmental duplication of the *AHLs*.

Gene duplication events occur with very high frequency in the genomes of all organisms (Lynch and Conery, 2000). We analyzed the collinear relationship between *B. napus* and *Arabidopsis*, *B. napus* and *B. rapa*, and *B. napus* and *B. oleracea*, respectively (Figure 5). The result revealed that there are a large number of homologous *AHLs* between *B. napus* and *Arabidopsis*, *B. napus* and *B. rapa*, *B. napus* and *B. oleracea*, respectively. Among them, *B. napus* and *B. rapa* have the most collinear genes (Figure 5). More than 78 percent of *BnaAHL* genes are generated by the whole genome duplication (WGD) or segmental duplication (Supplementary Table 2). Therefore, WGD or segmental duplication is a main driver of *BnaAHLs* expansion in the *B. napus* genome.

### *Cis*-Acting Element Analysis of *BnaAHLs*

*Cis*-acting elements regulate the initiation and efficiency of gene transcription by binding to transcription factors (Maire et al., 1989). We analyzed the *cis*-acting elements of 122 *AHLs* promoters with PlantCARE (Lescot et al., 2002; Table 2 and Supplementary Figure 2). Most *cis*-acting elements of *BnaAHLs* promoters are associated with plant growth and development, hormone response and abiotic stress response. Some *cis*-acting elements, namely, anaerobic induction, circadian control, defense and stress responsive, drought induction, light response, low temperature response, meristem expression, and wound response, are the main members in regulating environmental stress. Among them, the most common elements are associated with light response, indicating that the growth and development of plants regulated by BnaAHLs is affected by light. At the same time, we also found that the elements related to hormone response, including abscisic acid, MeJA, gibberellin and so on, accounted for a large proportion. These results indicate that *BnaAHLs* not only regulate plant growth and development, but also play important roles in hormone response and abiotic stress. This is consistent with the report that AHLs can not only regulate growth, but also activate stress response (Favero et al., 2020). The promoters of some duplicated gene pairs like *BnaAHL16b*/*BnaAHL16a*, *BnaAHL16g/BnaAHL16h*, and *BnaAHL13a*/*BnaAHL13b* contained basically the same *cis*-acting elements. The *cis*-acting elements of other duplicated gene pairs are not exactly the same, such as *BnaAHL22b*/*BnaAHL22a*, *BnaAHL23a*/*BnaAHL23b*, *BnaAHL13f*/*BnaAHL13g*. In general, these differences are mainly caused by the gain and loss of *cis*-acting elements, including MeJA responsive element, abscisic acid responsive element, defense and stress responsive element, circadian control element, and low temperature responsive element (Table 2, Supplementary Table 1, and Supplementary Figure 2). The *cis*-acting elements in the upstream promoter region of genes are closely related to the function of downstream genes (Chow et al., 2018). The diversity of *cis*-acting elements in these duplicated genes indicates that the responses of genes to biotic and abiotic stresses have changed, which is the molecular basis of gene functional diversity.

**TABLE 2:** The *cis*-acting elements on the *BnaAHLs* promoters.

		Type-I	Type-II	Type-III
Hormone-responsive elements	Abscisic-acid-responsive	137/56	53/38	42/28
	Auxin-responsive	35/56	23/38	13/28
	Gibberellin-responsive	45/56	38/38	10/28
	MeJA-responsive	98/56	82/38	46/28
	Salicylic-acid-responsive	34/56	24/38	14/28
	Zein-metabolism-regulation	22/56	10/38	7/28
Environmental stress-related elements	Anaerobic-induction	131/56	99/38	63/28
	Circadian-control	15/56	7/38	5/28
	Defense-and-stress-responsive	28/56	34/38	14/28
	Drought-inducibility	40/56	27/38	21/28
	Light-responsive	699/56	411/38	279/28
	Low-temperature-responsive	35/56	36/38	19/28
	Meristem-expression	19/56	11/38	13/28
	Wound-responsive	1/56	0/38	3/28

### Positive Selection in the *AHL* Gene Family

**FIGURE 6:**
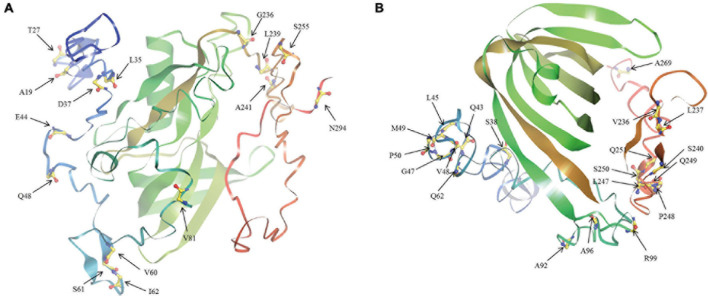
The tertiary structure of BnaAHL proteins. This is a schematic diagram of the tertiary spatial structure of the BnaAHL13j **(A)** and BnaAHL13g **(B)**, which are the Type-II and Type-III protein query sequence, respectively. The arrow points to the amino acid of positive selection.

The ratio of non-synonymous substitution rate to synonymous substitution rate, namely *Ka/Ks*, is a method for diagnosing sequence evolution (Sueoka, 1992; Hurst, 2002). *Ka/Ks* = 1 means that the gene is subject to neutral selection. *Ka/Ks* > 1 is considered to have a positive selection effect, otherwise it means that the gene is subject to purify selection. We calculated the *Ka/Ks* ratio of the *BnaAHLs* to study their evolutionary forms (Table 3). According to the phylogenetic tree, we first align each evolutionary branch separately, and then use four evolution models [M8 (ωs ≥ 1), M8a (ωs = 1), M7 (beta), and M5 (gamma)] to calculate the *Ka/Ks* value of each codon. The results showed that the *Ka/Ks* value of the three *AHL* types were all less than 0.5, indicating that the *AHL* gene family had undergone strong purification selection during the evolutionary process. The *Ka/Ks* value of Type-I AHLs is the smallest, ranging from 0.28 to 0.33. While the *Ka/Ks* value of Type-III is the largest, ranging from 0.42 to 0.51. It suggested that the purification effect of Type-I *AHLs* is stronger than those of the other two groups. We also find that some sites are subject to positive selection in the Type-II and Type-III groups predicted by M5 model. The results indicate that these positive selection sites are not in the conserved domains or motifs, but mainly in the random coils of the protein (Figure 6).

**TABLE 3:** Likelihood values and parameter estimation of positive selection among each codon of *BnaAHLs*.

Gene branches	Selection models	Ka/Ks	Log-likelihood	Positive-selection sites
Type-I	M8 (ωs ≥ 1)	0.296135	−21742.5	–
	M8a (ωs = 1)	0.292264	−21742.7	–
	M7 (beta)	0.287792	−21738.7	–
	M5 (gamma)	0.321483	−21784.1	–
Type-II	M8 (ωs ≥ 1)	0.397389	−20305.5	–
	M8a (ωs = 1)	0.394706	−20305	–
	M7 (beta)	0.39373	−20307.9	–
	M5 (gamma)	0.443497	−20343.6	A19/T27/L35/D37/E44/Q48/V60/S61/I62/V81/G236/L239/A241/S255/N294
Type-III	M8 (ωs ≥ 1)	0.431197	−17433	–
	M8a (ωs = 1)	0.424703	−17431	–
	M7 (beta)	0.448602	−17438.2	–
	M5 (gamma)	0.50116	−17448.4	S38/Q43/L45/G47/V48/M49/P50/Q62/A92/A96/R99/V236/L237/S240/L247/P248/Q249/S250/Q251/A269

### Analysis of Differential Expression of *BnaAHLs*

**FIGURE 7:**
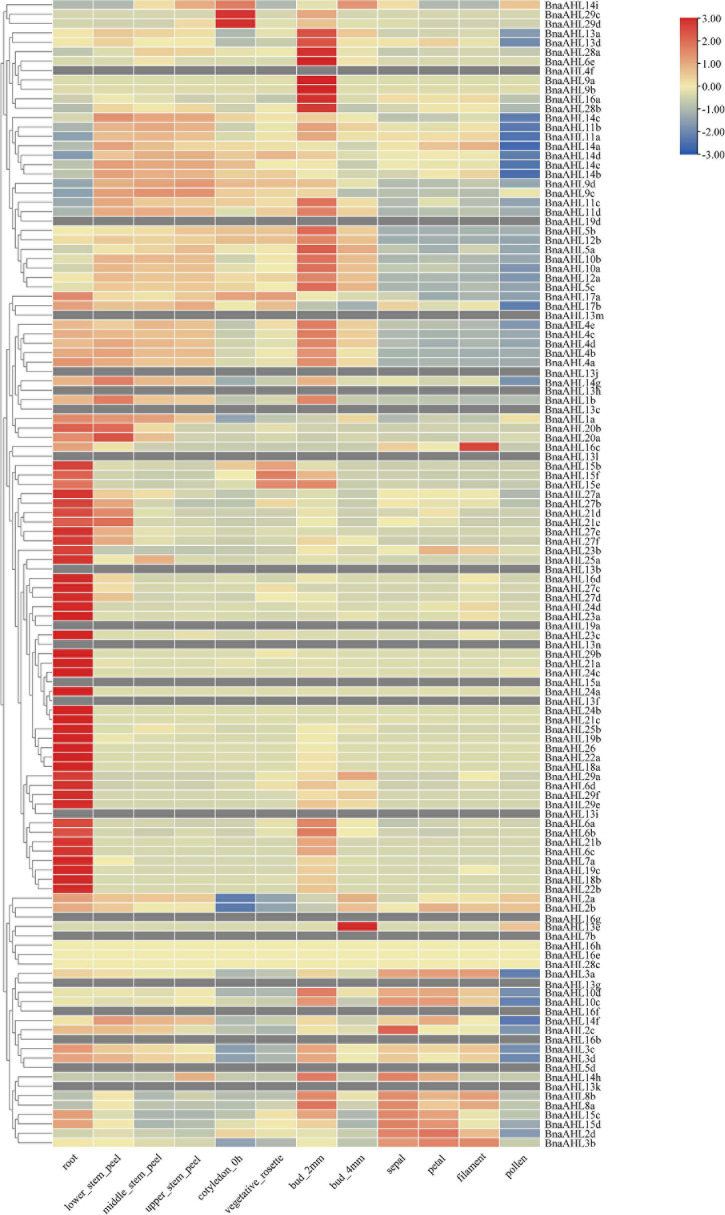
Expression patterns of *BnaAHLs* in different tissues. *BnaAHLs* are clustered by hierarchical clustering. The FPKM (the reads per kilobase per million mapped reads) method was used to normalize gene expression. The legend label is shown on the right side of the figure. Genes with high expression levels are shown in red, genes with ground expression levels are shown in blue. The gray box indicates that the expression level of the gene has not been found in the database.

Studies have revealed that the *AHL* genes not only play significant roles in the growth and development of plants (Zhao et al., 2014), but also help plants to resist some biotic and abiotic stresses (Jin et al., 2011). We obtained RNA-seq data from *B. napus* database (see text footnote 13), and used TBtools (Chen et al., 2020) to analyze the expression patterns of the *BnaAHLs* in different tissues, including root, stem, cotyledon, vegetative rosette, bud, sepal, petal, filament and pollen (Figure 7). RNA-seq data of 20 *BnaAHLs* were not found in the database. On the whole, expression level of most *BnaAHLs* is up-regulated in the root and pollen, but down-regulated in the bud (Figure 7). Expression level of about 30% *BnaAHLs* is up-regulated in the stem. In sepal, petal and filament, about 12 and 14% of *BnaAHLs* are highly expressed and low expressed, respectively. The results suggested that BnaAHLs could regulate a variety of developmental processes, which is consistent with the reported literatures (Ng et al., 2009; Karami et al., 2020; Sirl et al., 2020).

**FIGURE 8:**
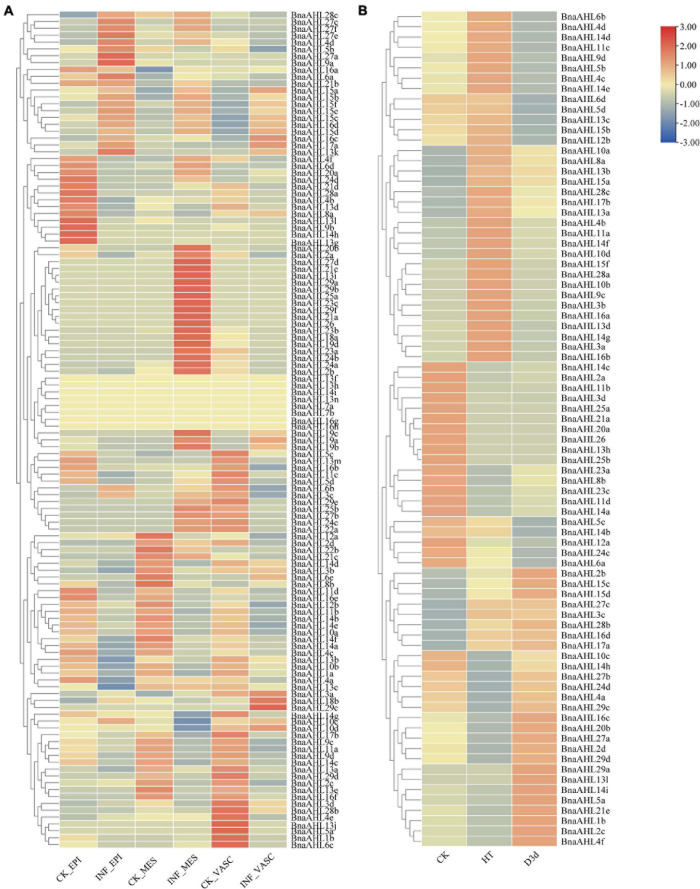
Expression patterns of *BnaAHLs* under infection by *S. sclerotiorum* **(A)** and under abiotic stresses **(B)**. *BnaAHLs* are clustered by hierarchical clustering. The TPM (Transcripts Per Million) method was used to normalize gene expression. The legend label is shown on the right side of the figure. Genes with high expression levels are shown in red, and genes with low expression levels are shown in blue. CK is control check, INF is the sample infected with *S. sclerotiorum*, EPI, MES, and VASC is epidermis, mesophyll and vascular, respectively. HT and D3d represent the expression of *B. napus* under heat treatment and drought treatment after 3 days, respectively.

Previous studies have shown that the *AHL*s have a response to some biological stresses (Lu et al., 2010; Kumar et al., 2018). In order to explore the biological functions of *BnaAHLs* under biological stress, we inspected the expression patterns of *BnaAHLs* based on the RNA-seq data infected by *S. sclerotiorum* (Figure 8A). Compared with the control group, the expression level of 10 *BnaAHLs* were up-regulated, while the expression pattern of 24 *BnaAHLs* were down-regulated in the epidermis, mesophyll and vascular of the leaf infected with *S. sclerotiorum*. 22 *BnaAHLs* are only highly expressed in mesophyll cells. These expression patterns indicate that *BnaAHLs* play important roles in regulating some biological stress.

We also investigated the expression patterns of *BnaAHLs* under drought and heat stress (Figure 8B). Expression levels of 14 *BnaAHLs* are up-regulated under drought stress, while the expression of 20 *BnaAHLs* are down-regulated under drought stress. Similarly, the 14 *BnaAHLs* whose expression levels were up-regulated under drought stress were also highly expressed under heat stress, while the 20 *BnaAHLs* that were down-regulated under drought stress were equally low expressed under heat stress. 34 *BnaAHLs* were highly expressed under heat stress, and 13 *BnaAHLs* were lowly expressed under drought stress. At the same time, the expression levels of some *BnaAHLs* (*BnaAHL10c*, *BnaAHL14h*, *BnaAHL27b*, *BnaAHL24d*, *BnaAHL4a*, *BnaAHL29c*, *BnaAHL16c*, *Bna AHL20b*, *BnaAHL27a*, *BnaAHL2d*, *BnaAHL29d*, *BnaAHL29a*, *BnaAHL13l*, *BnaAHL14i*, *BnaAHL5a*, *BnaAHL21e*, *BnaAHL1b*, *BnaAHL2c*, and *BnaAHL4f*) decreased under heat stress, but increased under drought stress. It suggests that the *AHL*s play important roles in the adaptation of rapeseed to drought and heat stress, which can also help *B. napus* to survive in the harsh environments. Therefore, *AHLs* not only play important roles in plant growth and development, but also can resist various biological and abiotic stresses (Zhou et al., 2016).

### Protein-Protein Interaction Analysis of *BnaAHLs*

**FIGURE 9:**
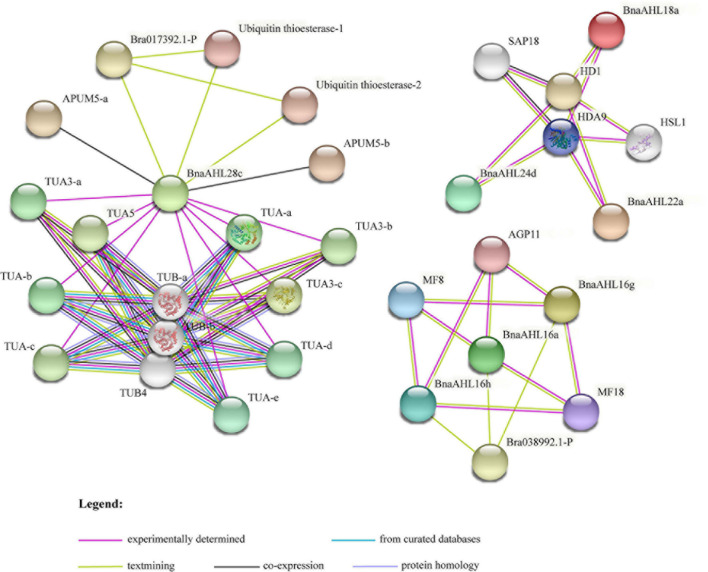
The interaction network of BnaAHL proteins. The line color indicates the type of interaction evidence. The legend is at the bottom of the figure.

Protein-protein interaction network can be used to analyze the complex life activities in cells according to the specific functions and processes of specific sub-networks (Hao et al., 2016). To better understand the functional roles of BnaAHLs, STRING (Szklarczyk et al., 2019) was used to investigate their interaction network. There are 7 members (BnaAHL28c, BnaAHL24d, BnaAHL18a, BnaAHL22a, BnaAHL16a, BnaAHL16h, and BnaAHL16g) of *BnaAHLs* involved in the construction of protein-protein interaction network. Some key proteins, like histone deacetylases (HDACs), arabinogalactan proteins (AGPs), α-tubulin (TUA), β-tubulin (TUB), ubiquitin thioesterase, arabidopsis pumilio (APUM), SIN3 ASSOCIATED POLYPEPTIDE P18 (SAP18), and HIGH-LEVEL EXPRESSION OF SUGAR-INDUCIBLE GENE2-LIKE1 (HSI2-LIKE1 or HSL1), were predicted to interact with these AHLs (Figure 9). HDACs can inhibit transcription by removing the acetyl groups in the tail core of histones and changing the chromatin structure (Hollender and Liu, 2008) and play key regulatory roles in plant development and stress response (Tian and Chen, 2001; Tian et al., 2003, 2005; Zhou et al., 2005; Chen et al., 2019). Research has demonstrated that AHL proteins can interact with HDACs to affect physiological processes such as flowering time (Yun et al., 2012). AGP6 and AGP11 are involved in a variety of signaling pathways (Costa et al., 2013) and developmental processes (Cheung et al., 1995; Willats and Knox, 1996; Suzuki et al., 2002; van Hengel and Roberts, 2002), especially pollen tube growth and pollen grain germination (Coimbra et al., 2010). AHL16 can directly regulate the expression of AGPs and activate the transcription of pollen development genes (Jia et al., 2015). Polymers of TUA and TUB guide the deposition of cellulose microfibrils during plant cell wall formation (Ledbetter and Porter, 1963). Studies have shown that TUA and TUB are specifically expressed in pollen (Carpenter et al., 1992), roots and leaves (Snustad et al., 1992; Cheng et al., 2001), and vegetative tissues (Kopczak et al., 1992; Abe et al., 2004). There is evidence that HSL1 can inhibit the ectopic expression of sugar-induced seed maturation genes during seed growth, and this protein play important roles in regulating seed germination (Tsukagoshi et al., 2007). As post-transcriptional regulators, APUM (Huh and Paek, 2014) and SAP18 (Song and Galbraith, 2006) were involved in some abiotic stress responses including salt stress. Here, some AHLs were predicted to interact with these proteins, indicating that AHL proteins, as a kind of transcription factor, are also involved in these plant growth, development and abiotic stress response.

## Discussion

The R-G-R-P amino acid core sequence was present in the AT-hook motif of *BnaAHLs*. R-G-R-P can prevent the change of DNA conformation by combining with the minor groove of DNA, thereby promoting the binding of transcription factors with the major groove (Huth et al., 1997). AT-hook can form a C-shaped structure, and the concave back side of the C-shaped structure can be inserted into the small groove of the B-form DNA to combine with the AT-rich DNA sequence (Huth et al., 1997). Among them, proline residues are responsible for maintaining the rigid connection of domains, while the Arg-Gly-Arg conserved sequence is mainly involved in DNA-protein interactions (Aravind and Landsman, 1998), thereby changing the chromatin structure (Strick and Laemmli, 1995). Although BnaAHL7b, BnaAHL14h, BnaAHL14i, BnaAHL21d, BnaAHL21e, and BnaAHL28c proteins lack the R-G-R-P conserved motif, they have the PPC/DUF296 domain. There are a motif 6 and a motif 7 in Type-II members. Type-I and Type-III members contain a motif 6 and motif 7, respectively. In Clade-B, motif 6 was lost and regained more than once during the evolutionary process, therefore the Type-II and Type-III AHL genes have been exchanged several times (Zhao et al., 2014). According to the distribution of phylogenetic tree and conserved motifs, *AHL* genes of Type-II and Type-III were not distributed separately, but crossed with each other. This finding further illustrates that the AHLs have undergone multiple transitions from Type-II to Type-III and from Type-III to Type-II. The emergence of new motifs may be caused by the insertion and deletion of nucleotides, but this mutation is deleterious. When duplicated genes are present, redundant copies can compensate for the loss of function caused by frameshift mutations, resulting in functional differentiation of genes (Litt and Irish, 2003; Vandenbussche et al., 2003; Raes and Van de Peer, 2005; Kramer et al., 2006; Litt, 2007; Shan et al., 2007). Part of type-II AHLs are formed by individual members of type-III AHL acquiring motif 6. A portion of type-II AHLs lost motif 6 to form type-III AHL (Zhao et al., 2014). R-G-R-P conserved sequence contained in motif 6 is responsible for binding to DNA, and each amino acid is responsible for a different function. A research has shown that the binding of motif 6 to DNA will be affected when the second R of mutation occurs (Zhao et al., 2013).

Study has shown that the intron-containing *AHLs* are diverged from intron-less *AHLs* (Zhao et al., 2014). Clade-A consist of the intron-less *AHLs*, while Clade-B is the opposite. This differential distribution of introns among different members exists not only in the *AHL* gene family but also in other gene families. In the *NADK* (Li et al., 2014) and *Sus* (Wang et al., 2015) gene family, the number, arrangement, and phase of introns are significantly different between subfamilies. Introns are ubiquitous in eukaryotic genomes. More and more evidences show that introns affect gene function by affecting gene expression regulation (Castillo-Davis et al., 2002). At the same time, introns can increase the recombination within and between genes and regulate the rate of gene evolution (Roy and Gilbert, 2006; Rogozin et al., 2012). In addition, many introns contain specific functional elements for exon shuffling and selective splicing, generic functions of non-coding DNA, and signaling as mRNA output from the nucleus (Fedorova and Fedorov, 2003; Le Hir et al., 2003). It is suggested that the *AHLs* of Clade-B in the rape genome have diverged from Clade-A. In the long evolutionary process, the acquisition of introns may cause the structural and functional changes of *BnaAHLs*. In addition, the motif structure, exon-intron pattern and amino acid sequence of *BnaAHLs* may be generated by gene duplication events during evolution. So far, there is no exact explanation for the appearance of introns. The scientific community has two main views on the development of introns. One view is that introns may be the result of tandem duplication within exons and expand one or more times in a manner similar to transposable factors (Zhuo et al., 2007). Another view is that introns emerge through reverse splicing catalyzed by the splicing mechanism itself (Coghlan and Wolfe, 2004). The rate of nucleotide substitution between genes is usually higher than that between introns (Gaffney and Keightley, 2006). Studies have shown that selective pressure on introns play protective roles in genes (Majewski and Ott, 2002). The selective pressure was negatively correlated with the number of introns (Gaffney and Keightley, 2006). Introns are subject to less selection pressure than exons, so the gains and losses of intron sequences are higher than those of exons, and their sequences differentiate more quickly than exons. *AHLs* of Type-III are subject to less selective pressure due to the presence of introns, so it can be reasonably explained that the value of *Ka/Ks* is greater than the value of Type-I *AHLs*.

Although the lengths of ChrC04 and ChrA04 chromosomes are significantly different, the *AHLs* on these two chromosomes are collinear. Therefore, these two chromosomes are homologous. We speculate that the unequal length of these two chromosomes may be caused by different chromosome duplication. The number of *BnaAHL* on ChrA06 and ChrC07 is significantly higher than that of ChrC06 and ChrA07, respectively. There was a significant collinear relationship between ChrA07 and ChrC06 of *B. napus* genome, which may be caused by chromosomal rearrangement. However, the collinearity between ChrA06 and ChrC07 chromosomes is very low. *AHL* genes exist on these two chromosomes in the form of gene clusters, which may be caused by tandem duplication after transposition.

After the separation of *Arabidopsis* and Brassica, one ancestor species similar with *Arabidopsis* formed hexaploid by triploidization. This hexaploid ancestor diverged into *B. rapa*, *B. oleracea*, and *B. nigra* (Lysak et al., 2005; Cheng et al., 2013; Chalhoub et al., 2014). *Brassica rapus* is a heterotetraploid formed by crossing *B. rapa* and *B. oleracea* (Chalhoub et al., 2014). The *Arabidopsis* genome contains 29 *AHLs*. Theoretically, the *B. napus* genome should contain nearly about 174 *AHLs*, but only 122 *AHLs* have been identified. These results suggest that the some *AHLs* were lost on a large scale after the triploidization.

The tertiary structure prediction and selective pressure analysis showed that there were positive selective amino acid sites near the AT-hook motif. The extended AT-hook motif (eAT-hook) has a stronger binding affinity to RNA than with DNA, which length is about 3 times of the ordinary AT-hook motif, and the basic amino acids are located about 12–15 residue distance on both sides of the G-R-P core sequences (Filarsky et al., 2015). The positive selection sites V81 of Type-II group and Q62 and A92 of Type-III group are within this range. The change at these sites may cause the AT-hook loss of binding activity with DNA, but increase the ability to bind to RNA.

Drought and heat are two important factors that affecting plant growth and development. For a long time, plants have evolved a variety of survival mechanisms in response to adversity, such as developing specific morphological traits and regulating metabolic pathways (Vijayaraghavareddy et al., 2020). Abiotic stress changes plant physiological processes by affecting gene expression, RNA or protein stability, and ion transport, etc. (Kosova et al., 2015). The first plant organ to sense salt stress is the root, which immediately relays this signal to the leaves through the stem, causing stomata to close and reducing water to loss (Cosgrove and Hedrich, 1991; Christmann et al., 2013). The analysis of expression patterns and *cis*-acting elements showed that *BnaAHLs* were highly expressed in root, and the upstream of *BnaAHLs cis*-acting elements were also involved in the regulation of light, drought, temperature, oxygen and other stresses. In addition, plant hormones also play roles in regulating plant growth and development by sensing drought and other environmental stress and transmitting signals (Lymperopoulos et al., 2018). The upstream of *BnaAHLs* carries a large number of *cis*-acting elements, including abscisic acid and MeJA *cis*-acting elements.

The expression patterns of some duplicated genes, including *BnaAHL10a*/*BnaAHL10b*, *BnaAHL9c*/*BnaAHL9d*, and *BnaAHL15c*/*BnaAHL15d*, were consistent in various tissues and under stress of heat, drought, and *S. sclerotiorum* (Figures 7, 8 and Supplementary Table 1). For the duplicated gene pair *BnaAHL1a*/*BnaAHL1b*, the expression pattern of the former is down-regulated in bud_2mm, and up-regulated in bud_4mm and pollen, while the expression pattern of the latter is completely opposite (Figure 7). Others examples like *BnaAHL4a*/*BnaAHL4b*, *BnaAHL10c*/*BnaAHL10d*, *BnaAHL11b*/*BnaAHL11a*, *BnaAHL11d*/*BnaAHL11c*, and *BnaAHL8b*/*BnaAHL8a*, their expression patterns are also different under heat stress. In addition, the expression pattern of *BnaAHL5a*/*BnaAHL5b* and *BnaAHL3c*/*BnaAHL3d* gene pairs was significantly different. Immune receptors in the cell membrane and cytoplasm of plants are the main innate immune systems. The plasma membrane-embedded PRRs (Pattern Recognition Receptors) have evolved to detect microbial PAMPs and activate PTI (Akira et al., 2006; Chisholm et al., 2006; Boutrot and Zipfel, 2017; Tang et al., 2017; Albert et al., 2020). *Sclerotinia sclerotiorum* is a destructive pathogen that can infect many monocotyledonous and dicotyledonous plants. Our study showed that the expression of *BnaAHLs* was significantly altered in *B. napus* leaves after *S. sclerotiorum* infecting. Duplication genes with opposite expression patterns were also found in leafs under the stress of *S. sclerotiorum* (Figure 8). These different expression patterns may be caused by the changes of *cis*-acting elements upstream of the gene of allopolyploid formed by natural polyploidization and their adaptation to the environment. These changes are the result of many genetic variations occurring in allopolyploid genomes (Song et al., 1995).

## Conclusion

In this study, we identified 122 *AHLs* in the *B. napus* genome and systematically analyzed them. Based on the phylogenetic analysis, the *AHL* gene family is divided into three branches, each of which has the same gene structure and motif. About 98% of *BnaAHLs* products were located in the nucleus. Collinearity analysis shows that the AHL gene family is relatively conserved in evolution of *B. rapa*, *B. oleracea*, *Arabidopsis*, and *B. napus*. *BnaAHLs* has high homology with *AHLs* of *B. rapa*, *B. oleracea*, and *Arabidopsis*. WGD duplication is the main method of *BnaAHLs* amplification. The *AHL* gene family has been subjected to purification or neutral selection during the evolution process, and the *BnaAHLs* of Type-I has the strongest purification effect. The *cis*-acting elements that regulate plant growth and development and assist plants to resist biotic and abiotic stresses are found in the BnaAHL promoters. This is also verified in tissue expression analysis. Some important BnaAHLs interacting proteins are found in the protein-protein interaction network, which indirectly indicates that *AHL* genes play important roles in plant growth and development. The identification and characterization of the *AHL* gene family in *B. napus* will contribute to further understand their role in these important oil crops.

## Data Availability Statement

The original contributions presented in the study are included in the article/Supplementary Material, further inquiries can be directed to the corresponding author/s.

## Author Contributions

W-MZ was the executor of the study, mainly completing the basic analysis of data, and writing the first draft of the manuscript. DF and X-ZC participated in the writing and revision of the manuscript. JC and X-LT were the conceitor and principal of the project, directed the research design and final correction of the data analysis results, and was responsible for the writing and revision of the thesis. All authors have read and agreed to the final text.

## Conflict of Interest

The authors declare that the research was conducted in the absence of any commercial or financial relationships that could be construed as a potential conflict of interest.

## Publisher’s Note

All claims expressed in this article are solely those of the authors and do not necessarily represent those of their affiliated organizations, or those of the publisher, the editors and the reviewers. Any product that may be evaluated in this article, or claim that may be made by its manufacturer, is not guaranteed or endorsed by the publisher.

## Footnotes

1
https://www.genoscope.cns.fr/brassicanapus/

2
https://www.arabidopsis.org/

3
https://web.expasy.org/protparam/

4
http://cello.life.nctu.edu.tw/

5
https://www.megasoftware.net/

6
http://meme-suite.org/tools/meme

7
https://www.ncbi.nlm.nih.gov/Structure/bwrpsb/bwrpsb.cgi

8
https://github.com/CJ-Chen/TBtools/releases

9
http://plants.ensembl.org/index.html

10
http://bioinformatics.psb.ugent.be/webtools/plantcare/html/

11
http://selecton.tau.ac.il/

12
https://zhanglab.ccmb.med.umich.edu/I-TASSER/

13
http://yanglab.hzau.edu.cn/BnTIR

14
https://www.ncbi.nlm.nih.gov/geo

15
https://string-db.org/cgi/input.pl
