# Supplementary figures and images for: Insights Into the Molecular Evolution of AT-Hook Motif Nuclear Localization Genes in Brassica napus
Source: Front Plant Sci. 2021 Sep 9;12:714305. doi: 10.3389/fpls.2021.714305 (PMC8458767; doi:10.3389/fpls.2021.714305)

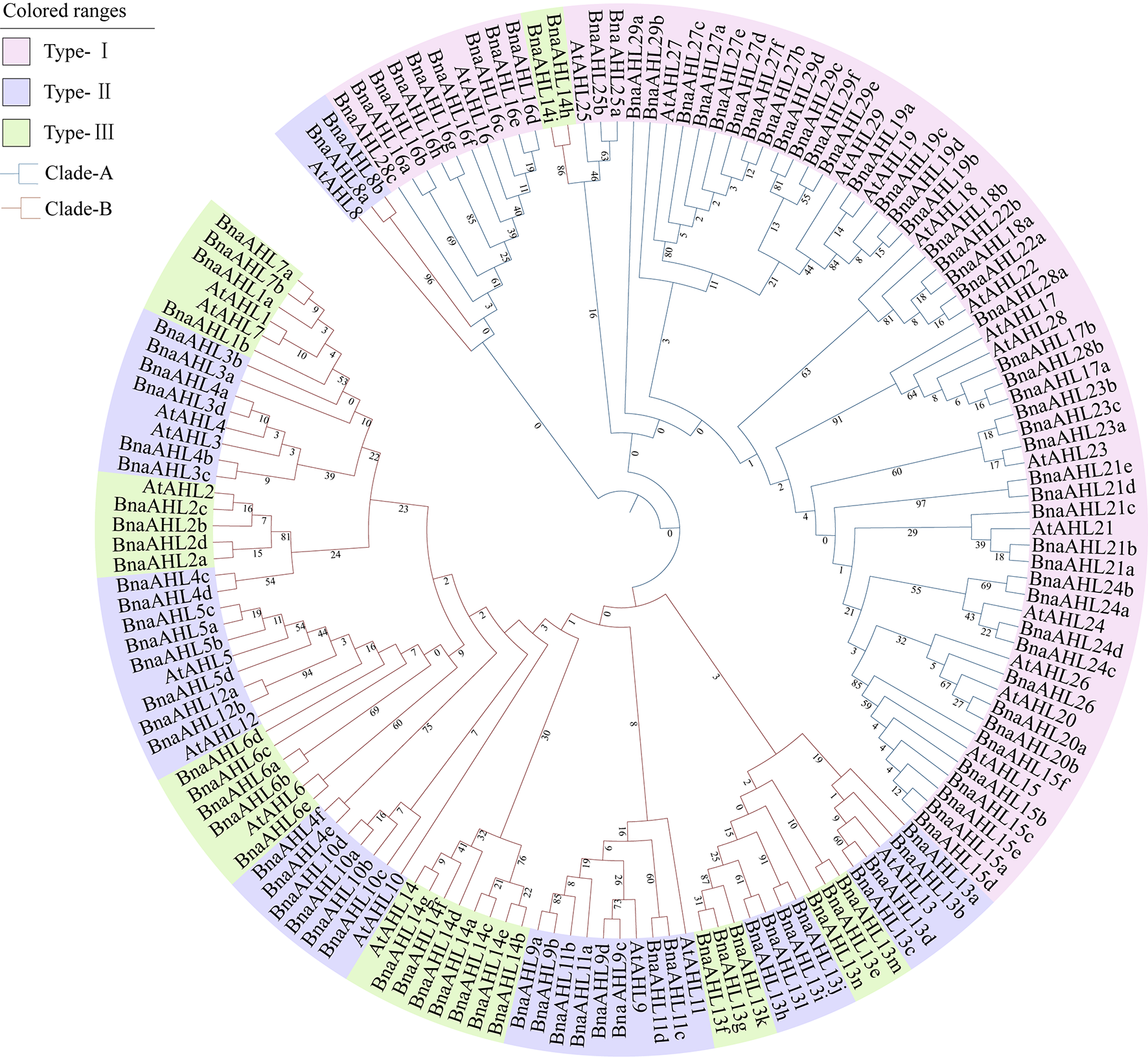

Supplement: Supplementary file 1 [file Image_1.TIF]

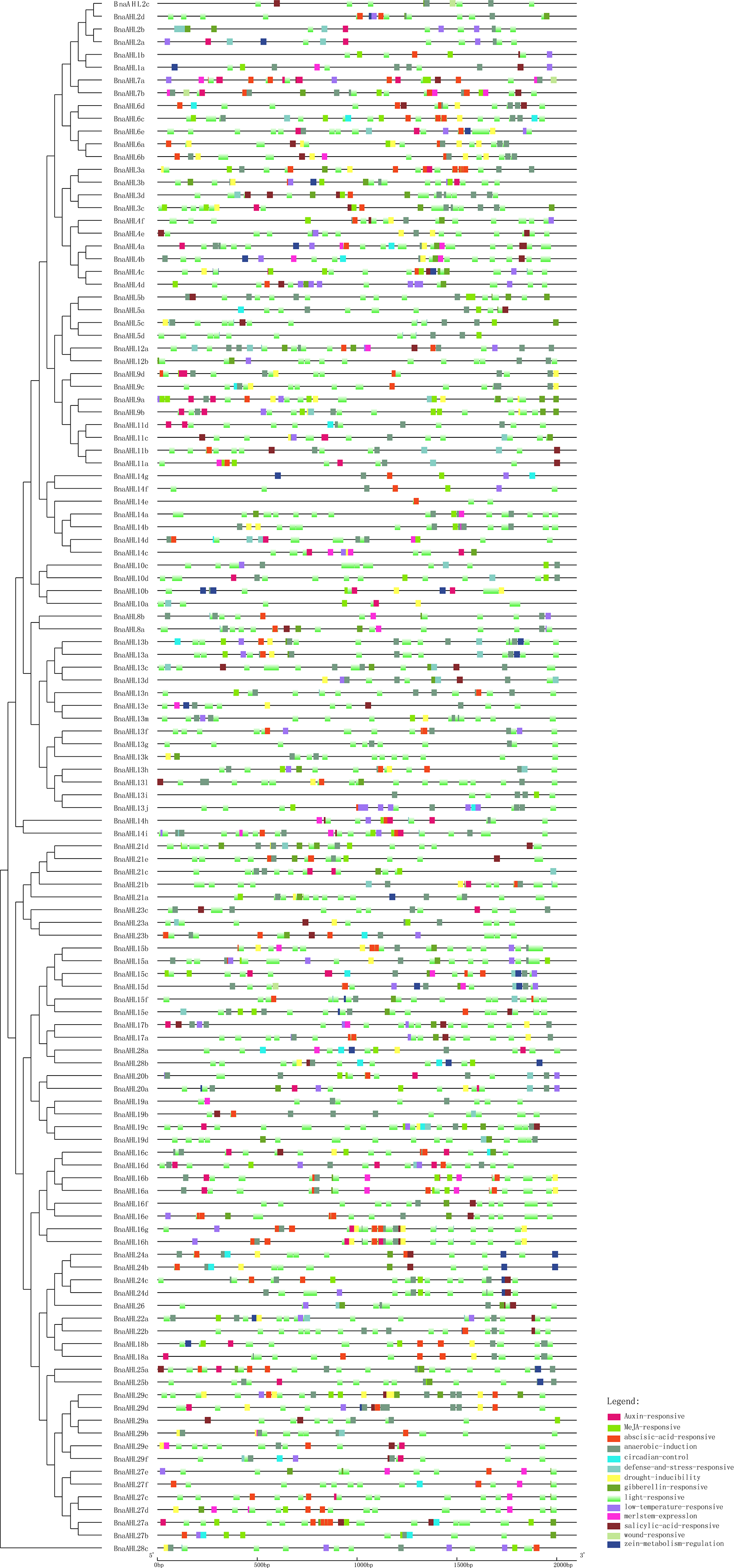

Supplement: Supplementary file 2 [file Image_2.TIF]
